# Intercellular Odontoblast Communication via ATP Mediated by Pannexin-1 Channel and Phospholipase C-coupled Receptor Activation

**DOI:** 10.3389/fphys.2015.00326

**Published:** 2015-11-10

**Authors:** Masaki Sato, Tadashi Furuya, Maki Kimura, Yuki Kojima, Masakazu Tazaki, Toru Sato, Yoshiyuki Shibukawa

**Affiliations:** ^1^Department of Physiology, Tokyo Dental CollegeTokyo, Japan; ^2^Department of Crown and Bridge Prosthodontics, Tokyo Dental CollegeTokyo, Japan

**Keywords:** odontoblast, transient receptor potential channel, pannexin-1, adenosine triphosphate, P2Y, paracrine

## Abstract

Extracellular ATP released via pannexin-1 channels, in response to the activation of mechanosensitive-TRP channels during odontoblast mechanical stimulation, mediates intercellular communication among odontoblasts in dental pulp slice preparation dissected from rat incisor. Recently, odontoblast cell lines, such as mouse odontoblast lineage cells, have been widely used to investigate physiological/pathological cellular functions. To clarify whether the odontoblast cell lines also communicate with each other by diffusible chemical substance(s), we investigated the chemical intercellular communication among cells from mouse odontoblast cell lines following mechanical stimulation. A single cell was stimulated using a glass pipette filled with standard extracellular solution. We measured intracellular free Ca^2+^ concentration ([Ca^2+^]_i_) by fura-2 in stimulated cells, as well as in cells located nearby. Direct mechanical stimulation to a single odontoblast increased [Ca^2+^]_i_, which showed sensitivity to capsazepine. In addition, we observed increases in [Ca^2+^]_i_ not only in the mechanically stimulated odontoblast, but also in nearby odontoblasts. We could observe mechanical stimulation-induced increase in [Ca^2+^]_i_ in a stimulated human embryo kidney (HEK) 293 cell, but not in nearby HEK293 cells. The increase in [Ca^2+^]_i_ in nearby odontoblasts, but not in the stimulated odontoblast, was inhibited by adenosine triphosphate (ATP) release channel (pannexin-1) inhibitor in a concentration- and spatial-dependent manner. Moreover, in the presence of phospholipase C (PLC) inhibitor, the increase in [Ca^2+^]_i_ in nearby odontoblasts, following mechanical stimulation of a single odontoblast, was abolished. We could record some inward currents evoked from odontoblasts near the stimulated odontoblast, but the currents were observed in only 4.8% of the recorded odontoblasts. The results of this study showed that ATP is released via pannexin-1, from a mechanically stimulated odontoblast, which transmits a signal to nearby odontoblasts by predominant activation of PLC-coupled nucleotide receptors.

## Introduction

Odontoblasts are dentin-forming cells that secrete dentin matrix proteins during physiological and pathological tooth formation. In the pathological setting, such as an enamel lesion, reactionary dentin is formed by various external stimuli applied to the dentin surface. The thermal, chemical, mechanical, and osmotic stimuli applied to the exposed dentin surface increase the hydrodynamic force and the velocity of dentinal fluid movement inside dentinal tubules (Andrew and Matthews, 2000; Charoenlarp et al., 2007). This increase in fluid movement induces deformation of plasma membrane of the odontoblast processes within the dentinal tubules (Magloire et al., 2010; Lin et al., 2011). Recent studies have indicated that odontoblasts express mechanosensitive ionic channels such as transient receptor potential (TRP) channel-vanilloid subfamily member-1, -2, and -4 (TRPV1, TRPV2, TRPV4) and TRP-ankyrin subfamily member-1 (TRPA1) (Magloire et al., 2010; Sato et al., 2013; Tsumura et al., 2013). Odontoblast cell membrane deformation activates various mechanosensitive-TRP channels as mechanosensors (Son et al., 2009; Tsumura et al., 2012; Shibukawa et al., 2015).

We have previously reported functional expression of G-protein- and phospholipase C (PLC)-coupling nucleotide receptor in odontoblasts (Shibukawa and Suzuki, 2003). These receptors are responsible for receipt of extracellular ATP released not only by dental pulp tissue damage (Cook et al., 1997; Liu et al., 2015), but also from other cells in the dental pulp, so as to establish intercellular communication. Most recently, we have reported that extracellular ATP released via pannexin-1 channels, in response to the activation of mechanosensitive-TRP channels during odontoblast mechanical stimulation, mediates intercellular chemical communication between odontoblasts and trigeminal ganglion (TG) neurons, to drive the sensory transduction mechanism for dental pain. We refer to this mechanism as “odontoblast hydrodynamic receptor theory” (Shibukawa et al., 2015). In addition, odontoblasts also established intercellular communication with each other via ATP/ADP and their nucleotide receptors, P2Y_1_ and P2Y_12_, expressed on these cells. This previous research was conducted by using primary cultured TG neurons and odontoblasts. The odontoblasts were obtained from dental pulp slice preparation from rat tissues (Shibukawa et al., 2015). Although the dental pulp slice preparations have been well established (Okumura et al., 2005; Son et al., 2009; Magloire et al., 2010; Tsumura et al., 2010, 2012, 2013; Shibukawa et al., 2015), odontoblast cell lines, such as mouse odontoblast lineage cells (OLCs; Arany et al., 2006; Fujisawa et al., 2012) or human dental pulp cells with odontoblastic differentiation (HDPs; Kitagawa et al., 2007), have also been widely used to investigate physiological/pathological odontoblast cellular functions (Ichikawa et al., 2012; Sato et al., 2013). Cooperative cellular function via intercellular signal communication plays an important role in the formation of hard tissues, including teeth (Iwamoto et al., 2010). Therefore, to clarify whether the odontoblast cell lines communicate with each other by diffusible chemical substance(s), we investigated the intercellular odontoblast signal communication when direct and focal mechanical stimulation was applied to single living odontoblasts.

## Materials and methods

### Cell culture

Mouse odontoblast lineage cells (OLC) (Arany et al., 2006; Fujisawa et al., 2012) were cultured in an alpha-minimum essential medium containing 10% fetal bovine serum, 1% penicillin-streptomycin, and 1% fungizone (Life technologies, Carlsbad, CA, USA) at 37°C with 5% CO_2_. The cells were positive for various odontoblast representative transcripts of dentin sialophosphoprotein, dentin matrix protein-1, and nestin, generously provided by Dr. Masayuki Tokuda, Kagoshima University, Kagoshima, Japan. HEK293 cells were cultured in Dulbecco's modified Eagle's medium containing 10% fetal bovine serum, 1% penicillin-streptomycin, and 1% fungizone at 37°C with 5% CO_2_.

### Solutions and reagents

Standard extracellular solution (standard ECS) was composed of 135 mM NaCl, 5 mM KCl, 2.5 mM CaCl_2_, 0.5 mM MgCl_2_, 10 mM NaHCO_3_, 10 mM 4-(2-hydroxyethyl)-1-piperazineethanesulfonic acid (HEPES), and 10 mM glucose and pH was adjusted to 7.4 by tris (hydroxymethyl) aminomethane (Tris) (328.5 mOsm/L). For Ca^2+^-free ECS, we removed extracellular Ca^2+^ (0 mM) from the standard ECS. Mefloquine and U73122 were obtained from TOCRIS Cookson (Bristol, UK). Capsazepine was obtained from Wako pure chemical (Osaka, Japan). All stock solutions were prepared in dimethyl sulfoxide. These stock solutions were diluted with standard ECS to the appropriate concentration before use. Except where indicated, all reagents were obtained from Sigma Chemical Co. (St. Louis, MO, USA).

### Direct mechanical stimulation of a single odontoblast

Direct mechanical stimulation was applied using a fire-polished glass micropipette with a tip diameter of 2–3 μm. The stimulation micropipettes were pulled from glass pipette tubes (Harvard apparatus, UK) by using a DMZ Universal Puller (Zeitz instruments, Martinsried, Germany) and the micropipettes were filled with standard ECS. The micropipette was operated using a micromanipulator (NHW-3, Narishige, Tokyo, Japan). The micropipette was placed at a site just above the cell attachment position and was gently moved by 4.3, 8.5, or 12.8 μm in the vertically downward direction at 2.2 μm/s velocity to depress the cell membrane, to generate a focused mechanical stimulation.

### Measurement of peripheral cell length

Detached odontoblasts resuspended in standard ECS were placed into culture dishes at 37°C and 5% CO_2_ for 1 h. Images of odontoblasts in standard ECS were acquired using an intensified charge-coupled device camera (Hamamatsu Photonics, Shizuoka, Japan) mounted on a microscope (Olympus, Tokyo, Japan). The odontoblast radius was determined from the images obtained (AQUACOSMOS, HCImage, Hamamatsu Photonic, Shizuoka, Japan). Changes in peripheral cell length by mechanical stimulation were normalized to that found without any stimulation in the standard ECS.

### Measurement of Ca^2+^-sensitive dye fluorescence

Odontoblasts were incubated for 60 min (37°C) in standard ECS containing 10 μM fura-2 acetoxymethyl ester (Dojindo Laboratories, Kumamoto, Japan) and 0.1% (w/v) F-127 pluronic acid (Life technologies), followed by rinsing with fresh standard ECS. [Ca^2+^]_i_ was measured using an AQUACOSMOS and HCImage system with an excitation wavelength selector and an intensified charge-coupled device camera incorporated onto a microscope. Fura-2 fluorescence emission was measured at 510 nm in response to alternating excitation wavelengths of 340 nm (F340) and 380 nm (F380). [Ca^2+^]_i_ was measured as the fluorescence ratio (R_F340∕F380_) at two excitation wavelengths of 380 nm and 340 nm, and then expressed as *F/F*_0_ units; the R_F340∕F380_ value (*F*) was normalized to the resting value (*F*_0_).

### Whole-cell patch-clamp recording

Patch-clamp recordings of the whole-cell configuration were performed under voltage-clamp conditions. Patch pipettes (2–5 MΩ) were pulled from capillary tubes by using a DMZ Universal Puller (Zeitz Instruments, Martinsried, Germany), and the pipettes were filled with an intracellular solution. The intracellular solution contained 140 mM KCl, 10 mM NaCl, and 10 mM HEPES (pH adjusted to 7.2 by Tris). Whole-cell currents were measured using a patch-clamp amplifier (L/M-EPC-7+; Heka Elektronik, Lambrecht, Germany), and were monitored and stored using pCLAMP software (Molecular Devices, Sunnyvale, CA, USA), after digitizing the analog signals at 1 kHz (DigiData 1440A, Molecular Devices) and filtering the signals digitally at 100 Hz using pCLAMP. The data were analyzed offline by using pCLAMP and the technical graphics/analysis program ORIGIN (MicroCal Software, Northampton, MA, USA). All experiments were conducted at room temperature (30 ± 1.0°C).

### Statistical analysis

Data are expressed as the mean ± standard deviation (SD) or standard error (SE) of the mean of *N* observations, where *N* represents the number of tested cells or separate experiments, respectively. Statistical differences were evaluated using analysis of variance (ANOVA) and the Steel-Dwass *post-hoc* test, and *P* < 0.05 was considered to be significant.

## Results

### Direct mechanical stimulation induces Ca^2+^ influx in the odontoblasts

**Figure 1:**
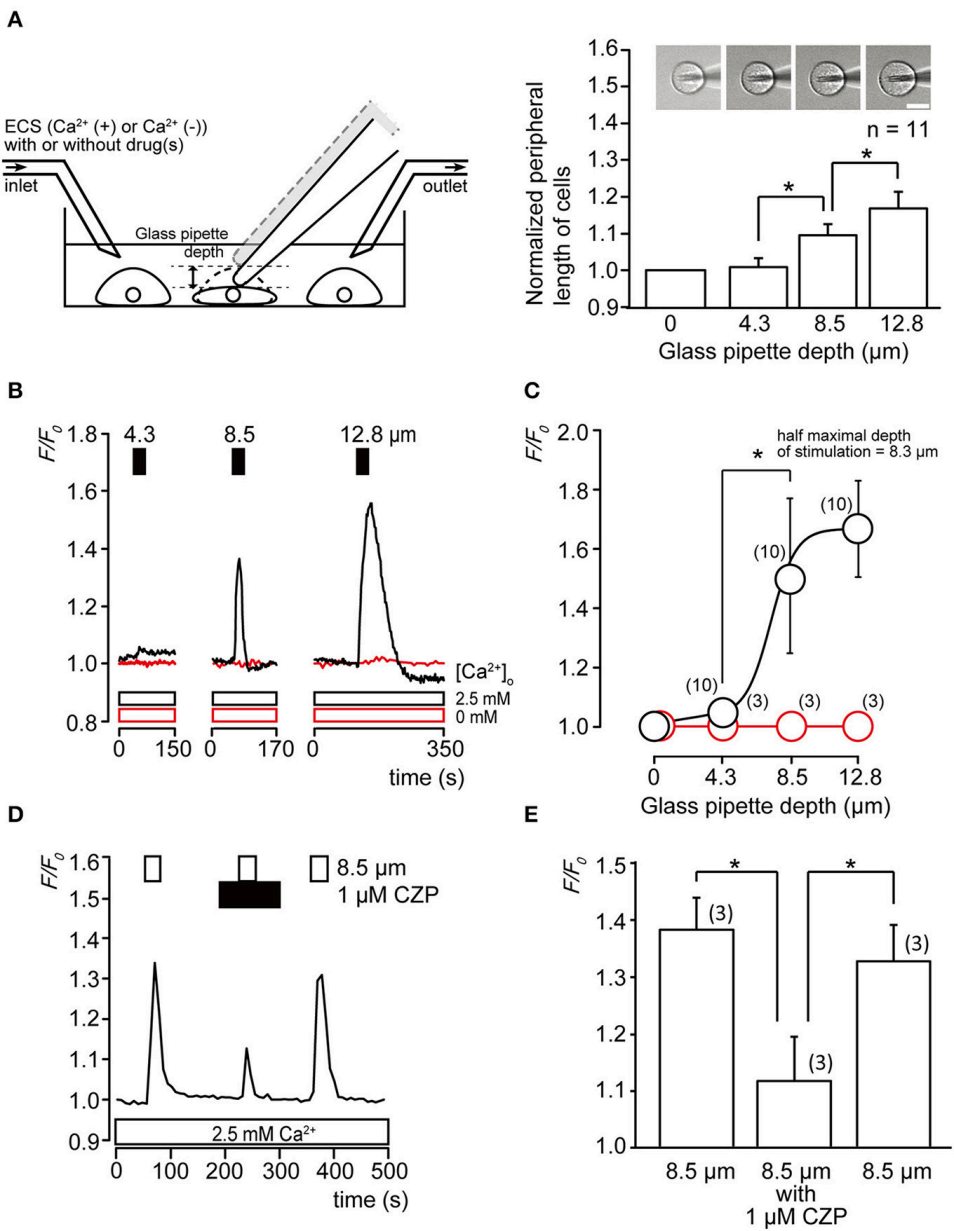
**Cell deformation by direct stimulation induces increase in [Ca^2+^]_i_ (A)** Single-cell direct mechanical stimulation with glass micropipette in the recording bath. The relationship between changes in the peripheral length of cells and intensity of mechanical stimulation in terms of depth reached by the pipette. Normalized cell size increased with increasing mechanical stimulation, each normalized cell size was 1.02 ± 0.03-fold by 4.3 μm, 1.10 ± 0.03-fold by 8.5 μm, and 1.16 ± 0.05-fold by 12.8 μm of the vertical downward displacement of the pipette. Bars represent mean ± SD of 11 cells. Statistically significant differences between values are indicated by asterisks. ^*^P < 0.05 (**A** right). Top panel shows images of cells during each stimulation. Bar indicates 10 μm. **(B)** Traces of transient increase in [Ca^2+^]_i_ during a series of mechanical stimulations induced by vertical micropipette displacements at depths of 4.3, 8.5, and 12.8 μm (upper filled boxes) in standard ECS with extracellular 2.5 mM Ca^2+^ (black lines and black line boxes) or without extracellular Ca^2+^ (0 mM) (red lines and red line boxes). **(C)** Linear plot of *F/F*_0_ value against direct mechanical stimulation intensity with extracellular 2.5 mM Ca^2+^ (black) or without extracellular Ca^2+^ (red). The numbers in parentheses indicate the number of tested cells. **(D)** Mechanical stimulation-induced Ca^2+^ influx was inhibited by TRPV1 antagonist (upper black box). [Ca^2+^]_i_ increase was elicited by 8.5 μm direct mechanical stimulation (upper white boxes). **(E)** Summary bar graphs of the increase in [Ca^2+^]_i_ induced by mechanical stimulation (to 8.5 μm) without (left column) or with (middle column) application of TRP channel antagonist. Recovery effect is shown in the right column. The resting value, *F/F*_0_ = 1.0. Each bar indicates the mean ± SD for three cells. Statistically significant differences between columns (shown by solid lines) are indicated by asterisks. ^*^P < 0.05.

Cell peripheral length was calculated to measure changes in cell size with or without exposure by penetration of a glass micropipette at three different depths (4.3, 8.5, and 12.8 μm). The normalized peripheral length of cells significantly increased as the glass pipette depth was measured from 0 to 8.5 μm (Figure 1A, *P* < 0.05), indicating that direct mechanical stimulation using a glass pipette induced stretching of the odontoblast plasma membrane. The peripheral length of cells without mechanical stimulation was found to be 46.2 ± 1.4 μm (*N*=11).

A series of direct mechanical stimulations (4.3–12.8 μm) elicited transient increases in intracellular free Ca^2+^ concentration ([Ca^2+^]_i_) (Figure 1B, black lines) in the presence of extracellular Ca^2+^, while in the absence of extracellular Ca^2+^ (Ca^2+^-free extracellular solution; Ca^2+^-free ECS), mechanical stimulation-induced [Ca^2+^]_i_ increases were completely abolished (Figure 1B, red line), indicating that mechanical stimulation of the odontoblasts elicited Ca^2+^ influx. The linear plot in Figure 1C shows *F/F*_0_ values as a function of applied direct mechanical stimulation. Dependence of [Ca^2+^]_i_ on mechanical stimulation by the pipette was confirmed by fitting the data to the following function:

(1) $$\begin{array}{r} \text{A}=\left({\text{A}}_{\text{min}}-{\text{A}}_{\text{max}}\right)∕\left({1+\text{e}}^{\left(\text{x}-K∕\text{dx}\right)}\right)+{\text{A}}_{\text{max}}\ldots \end{array}$$

where *K* is the half-maximal depth of a mechanical stimulation applied on the odontoblast (8.3 μm), A_max_ is maximal and A_min_ is minimal *F/F*_0_ response. The x indicates applied depth of the glass pipette.

### Nonselective TRPV1 channel antagonist inhibits direct mechanical stimulation–induced Ca^2+^ influx in odontoblast

To further investigate the Ca^2+^ influx pathway activated by direct mechanical stimulation, the effects of nonselective TRPV1 and TRP Melastatin subfamily member 8 (TRPM8) channel antagonist, capsazepine (CZP), on direct mechanical stimulation-induced Ca^2+^ influx were examined. In the presence of extracellular Ca^2+^ (2.5 mM), [Ca^2+^]_i_ in odontoblasts showed a rapid and transient increase during direct mechanical stimulation (8.5 μm), which was significantly and reversibly inhibited by 1 μM CZP to 32.4 ± 12.1% (*N*=3) (Figures 1D,E).

### Direct mechanical stimulation also induces [Ca^2+^]_i_ increase in odontoblasts around the stimulated odontoblast

**Figure 2:**
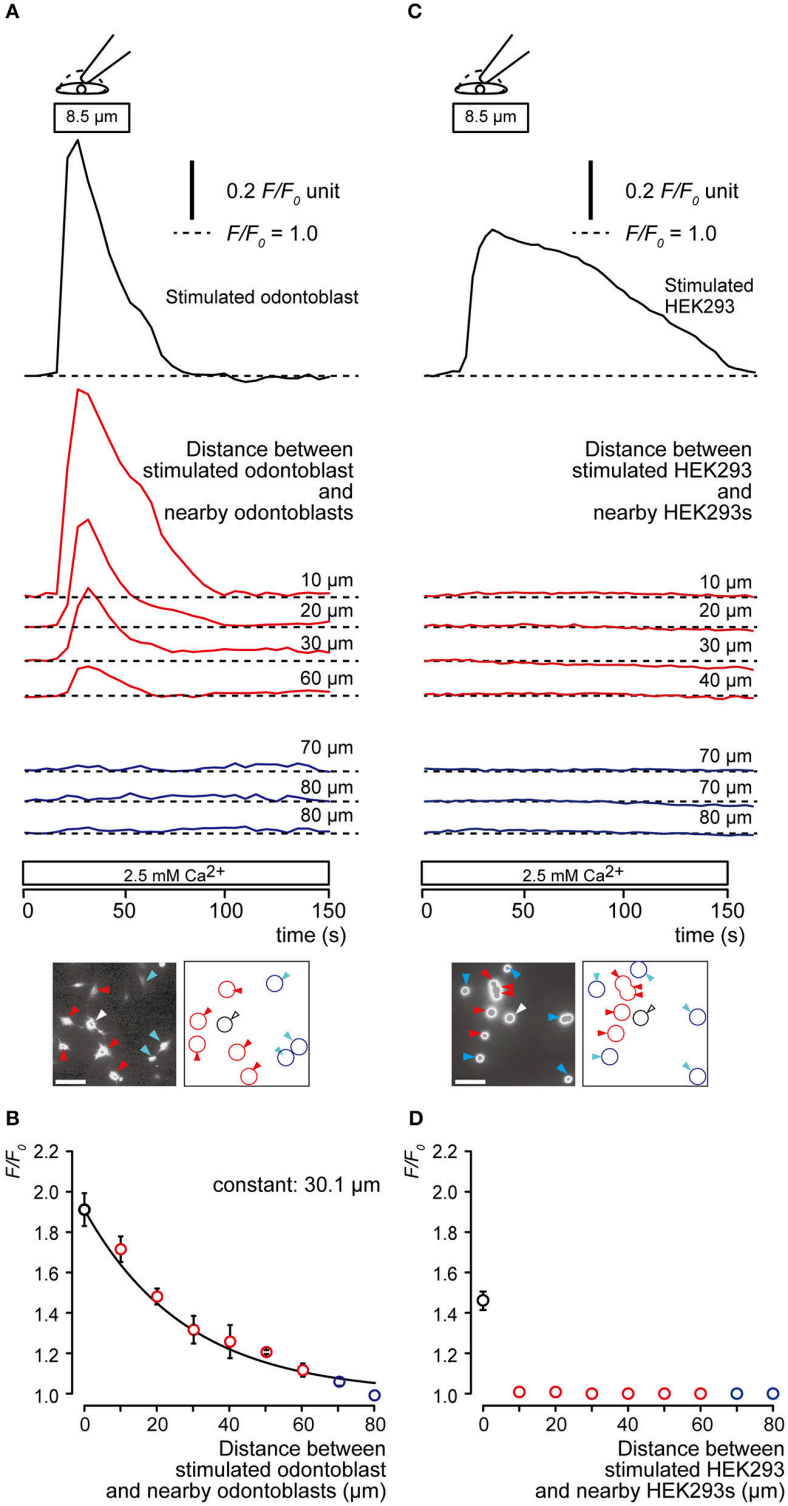
**Communication between a mechanical stimulated odontoblast and nearby odontoblasts. (A,C)** Representative traces showing transient increase in [Ca^2+^]_i_ from a mechanically stimulated cell (upper black traces), nearby (middle red traces) and distant cells (lower blue cells) in odontoblast **(A)** and HEK293 cell **(C)** during focal, direct mechanical stimulation of a single cell. Cell deformation was induced by displacement of a micropipette to a depth of 8.5 μm (upper open boxes and drawings) in a single cell in standard ECS (2.5 mM Ca^2+^). Bottom in **(A,C)** show images of relative fluorescence ratio (*F*_0_) before recordings (white bars show 50 μm), and their schematic representations. Stimulated cells are shown by white arrowhead and black lines, nearby cells are indicated by red arrowheads and red lines, and distant cells are represented by blue arrowheads and blue lines. Each horizontal dotted line shows base line (as *F/F*_0_ = 1.0) for each response. Responses from nearby (four red traces) or distant cells (three blue traces) were recorded 10–80 μm away from stimulated cells. **(B,D)** *F/F*_0_ values (data points) as a function of the distance from the stimulated cell (0 μm) to nearby (red symbols) or distant cells (blue symbols) for odontoblasts **(A)** and HEK293 cells **(D)**. The superimposed lines denote the best fit of a single exponential function. This analysis yielded spatial constants (τ) for the distance-dependent decrease in [Ca^2+^]_i_. The increase of [Ca^2+^]_i_ was observed in neither nearby nor distant HEK293 cells. Data points represent the mean ± SE from 3 to 32 cells in 3 separate experiments in **(B)** and 3 to 25 cells in 3 separate experiments in **(D)**.

We measured the increase in [Ca^2+^]_i_ by direct mechanical stimulation not only in the single odontoblast, but also in the neighboring odontoblasts. Direct mechanical stimulation of the single odontoblast increased [Ca^2+^]_i_ transiently. We also observed an increase in nearby odontoblasts (Figure 2A). The increase in [Ca^2+^]_i_ in nearby odontoblasts was reduced by increasing their distance from the stimulated odontoblast (Figure 2A). The plot in Figure 2B shows *F/F*_0_ values as a function of distance from stimulated odontoblast to nearby odontoblasts. The spatial constant (τ), was obtained by fitting the data to the exponential decay function. The spatial constant was 30.1 μm. Direct mechanical stimulation of a human embryonic kidney (HEK) 293 cell increased [Ca^2+^]_i_ transiently, whereas [Ca^2+^]_i_ increase in HEK293 cells near the stimulated cell was not observed (Figure 2C). The plot in Figure 2D shows *F/F*_0_ values as a function of distance from stimulated HEK293 cells to nearby HEK293 cells. The spatial constant could not be measured for HEK293 cells.

### Odontoblasts release ATP into the extracellular space via pannexin-1 channel and receive it via phospholipase C-coupled receptor

**Figure 3:**
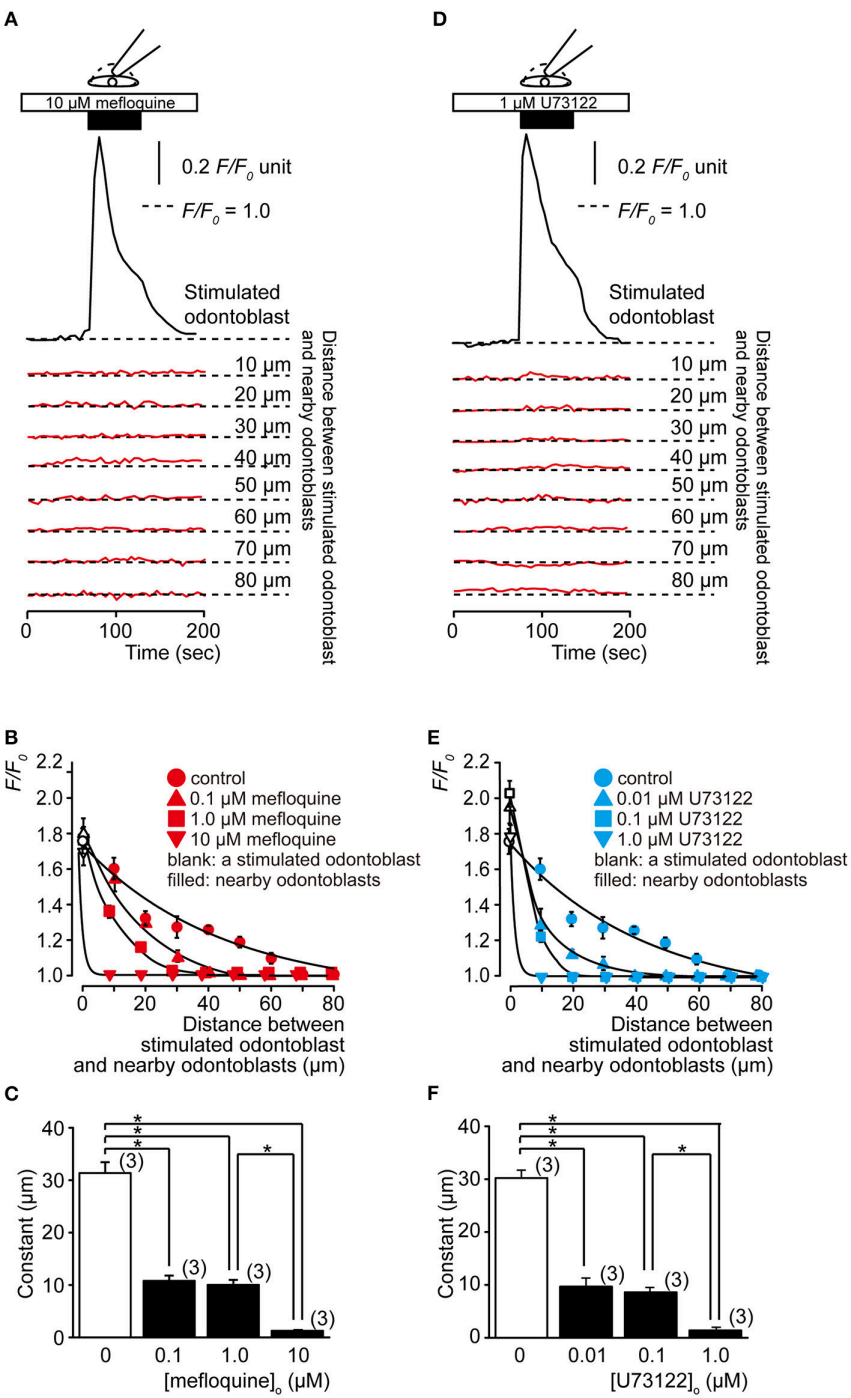
**Intercellular odontoblast communication via ATP**. Effects of pannexin-1 inhibitor, mefloquine, and phospholipase C (PLC) inhibitor, U73122, on the increase in [Ca^2+^]_i_ following focal and direct mechanical stimulation of a single odontoblast. **(A,D)** Transient increase in [Ca^2+^]_i_ from a mechanically stimulated odontoblast (upper black traces), nearby and distant odontoblasts (lower red traces), during single odontoblast stimulation in the presence of mefloquine **(A)** or U73122 **(D)**. Mechanical stimulation was applied by displacement of a micropipette to a depth of 8.5 μm (upper filled boxes and drawings) in standard ECS (2.5 mM Ca^2+^). Horizontal dotted lines show the baseline (*F/F*_0_ = 1.0) for each response. Responses from nearby and distant odontoblast were recorded from cells located 10 to 80 μm away from the stimulated odontoblast. This distance is indicated on the right side of each trace. **(B,E)** *F/F*_0_ values (data points) as a function of the distance from a stimulated odontoblast (0 μm) to nearby odontoblast, with or without pannexin-1 inhibitor (0.1–10 μM mefloquine; red symbols in **B**) and phospholipase C inhibitor (0.01–1 μM U73122; blue symbols in **E**). Each mechanical stimulation induced [Ca^2+^]_i_ increase in the stimulated odontoblast is shown by open symbols with each concentration of mefloquine or U73122. This response was not affected by treatment with mefloquine or U73122. The superimposed lines denote the best fit of a single exponential function. This analysis yielded spatial constants (τ) of the distance-dependent decreases in [Ca^2+^]_i_ (solid lines in **B,E**) with or without (filled circles) mefloquine **(B)** and U73122 **(E)**. Data points represent the mean ± SD from 3 to 41 cells in **(B,E)** from each three separate experiment. **(C,F)** Summary bar graphs of the mean τ values of the distance-dependent decrease in [Ca^2+^]_i_, measured from stimulated to nearby odontoblast, with pannexin-1 inhibitor **(C)** or phospholipase C inhibitor **(F)**. Each bar indicates mean ± SD. The numbers in parentheses in **(C,F)** indicate the number of tested cells. Statistically significant differences between spatial constant values are indicated by asterisks. ^*^P < 0.05.

Using 10 μM mefloquine, which is an inhibitor of the pannexin-1 channel, mechanical-stimulation-induced [Ca^2+^]_i_ increase was observed in stimulated odontoblast, whereas in nearby odontoblasts no significant increase in [Ca^2+^]_i_ was observed (Figure 3A). In addition, mefloquine (0.1, 1.0, and 10 μM) inhibited [Ca^2+^]_i_ response in nearby odontoblasts in a concentration- and spatial-dependent manner (Figures 3B,C). The administration of 1.0 μM U73122, an inhibitor of phospholipase C, significantly suppressed mechanical stimulation-induced increase in [Ca^2+^]_i_ in nearby odontoblasts, but not the stimulated cell (Figure 3D). U73122 (0.01, 0.1, and 1.0 μM) inhibited [Ca^2+^]_i_ responses in nearby odontoblasts in a concentration- and spatial-dependent manner (Figures 3E,F). The decrease in [Ca^2+^]_i_ in nearby odontoblasts with increasing distance from stimulated odontoblasts occurred rapidly (Figures 3B,E) in a dose-dependent manner on mefloquine (Figure 3C) and U73122 treatment (Figure 3F), showing significant decrease in spatial constants.

### Evoked inward currents in neighboring odontoblasts following mechanical stimulation of a single odontoblast

**Figure 4:**
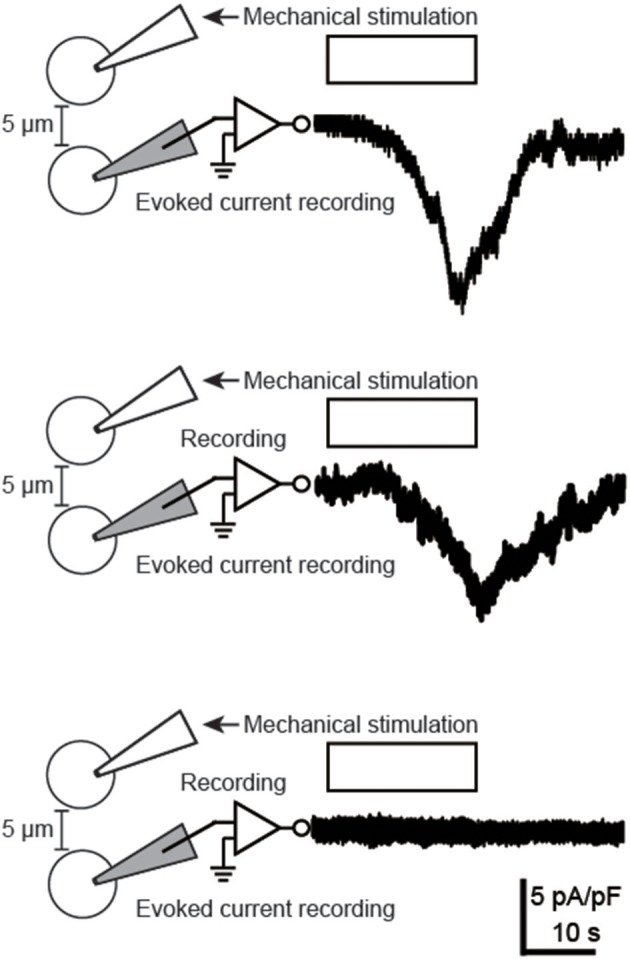
**Evoked inward currents in neighboring odontoblasts following mechanical stimulation of a single odontoblast**. Traces of inward currents (upper two traces) were recorded by using whole-cell patch-clamp recordings of the cells located 5 μm away from a stimulated odontoblast, following mechanical stimulation (the white boxes and the drawings on the left). Pictures of the whole-cell patch-clamp recording are also shown. Note that we could record evoked inward currents in only 2 out of 42 tested odontoblasts. Lower trace shows current from cell which did not elicit inward current, following mechanical stimulation. The recording was obtained from three separated cells.

The whole-cell patch-clamp method was used to record membrane currents that are activated by intercellular transmitter released from mechanically stimulated odontoblasts (Figure 4). We recorded an evoked inward current in only 2 out of 42 tested odontoblasts located 5 μm from the mechanically stimulated cell (in 42 experiments). These currents had delay times of 7–10 s in their activation.

## Discussion

In this study, we demonstrated that intercellular signal transduction between mechanically stimulated and neighboring odontoblasts occurs via activation of mechanosensitive TRP channels, release of ATP from the pannexin-1 channel, and activation of PLC-coupled receptors. Since we previously reported that TRPM8 channels are not expressed in mouse odontoblasts, CPZ-sensitive mechanical stimulation-induced [Ca^2+^]_i_ response in the present study is mediated by TRPV1 channels. Activation of TRPV1 channels, acting as sensor proteins (Magloire et al., 2010; Sato et al., 2013; Tsumura et al., 2013), induces the release of ATP via pannexin-1 channels from mechanically stimulated odontoblasts. ATP released from mechanically stimulated odontoblasts, as an intercellular transmitter in the extracellular medium, then activates PLC-coupled ATP/ADP receptors in neighboring odontoblasts. These results are in line with our previous results showing that mechanically stimulated odontoblasts release ATP via pannexin-1 channels to the neighboring odontoblasts, which activates the P2Y_1_ and P2Y_12_ receptors (Shibukawa et al., 2015).

The changes in the peripheral length and the increase in [Ca^2+^]_i_ depended on the intensity of the direct mechanical stimulation applied to odontoblasts. In the previous and present study, repeated mechanical stimulation to the odontoblasts induced increase in [Ca^2+^]_i_ repeatedly (Shibukawa et al., 2015). Although these results suggested that the stimuli did not induce any unfavorable effects to the cells, application of mechanical stimulation was limited to three times in the present study to avoid cell damage.

Treatment of the culture medium with an inhibitor of ATP-releasing pannexin-1 channels abolishes the increase in [Ca^2+^]_i_ in neighboring odontoblasts, but not in mechanically stimulated odontoblasts, indicating that ATP released via pannexin-1 acts as an intercellular transmitter for odontoblast–odontoblast chemical communication. In previous immunohistochemical studies, pannexin-1 was found to be localized throughout the cell body of odontoblasts (Shibukawa et al., 2015). In addition, TRPV4 and TRPA1 channel activation also elicits ATP release in odontoblasts (Egbuniwe et al., 2014). When direct mechanical stimulation was applied to HEK293 cells, internal Ca^2+^ was increased in the mechanically stimulated cells, but not in the neighboring cells. This is consistent with previous results showing that pannexin-1 is not expressed in HEK293 cells (Langlois et al., 2014), and suggests that intercellular communication via diffusible substances (i.e., intercellular transmitters) is established specifically in inter-odontoblast cellular networks in the dental pulp.

Odontoblasts express connexin (Cx) hemi-channels, forming a gap junction and establishing cell–cell communication (Goldberg et al., 1981; Sasaki et al., 1982; Callé, 1985; Goldberg and Sasaki, 1985; About et al., 2001; Muramatsu et al., 2004; Ikeda and Suda, 2013). Connexin 43, a gap junction protein, is expressed in odontoblasts (Ikeda and Suda, 2013; Muramatsu et al., 2013), and seems to play a role in electrical communication between odontoblasts. It has been reported that connexin 43 also releases ATP extracellularly in the pathological setting, such as low (almost 0 mM) extracellular Ca^2+^ circumstance or high (c.a. +60 mV) membrane potential. Since we did not expose the odontoblast to such extracellular conditions, contribution of connexin 43 to intercellular odontoblasts chemical communication is unlikely (Shibukawa et al., 2015).

Use of U73122, an inhibitor of phospholipase C, did not affect calcium response in the mechanically stimulated odontoblast, but abolish it in the neighboring odontoblasts. P2Y receptors are coupled with PLC and activated principally by endogenous nucleotides, ATP, and ADP (Burnstock, 2007). A specific enzyme, nucleoside triphosphate diphosphohydrolase-2 (NTPDase2) (Liu et al., 2012), is expressed in the Schwann cells of the dental pulp and/or the odontoblast membrane, and has been reported to hydrolyze extracellular ATP to ADP. Thus, both, released ATP and ADP from degradation of the ATP by NTPDase2, activate P2Y receptors in nearby odontoblasts. In addition, an ATP derivative, 2-methylthio-ATP, mobilizes [Ca^2+^]_i_ in the odontoblasts (Shibukawa and Suzuki, 2003), showing expression of the P2Y_1_ and P2Y_12_ receptors. We previously reported that P2Y_1_ and P2Y_12_ receptor activation in odontoblasts established inter-odontoblast communication (Shibukawa et al., 2015).

On the other hand, as shown in Figure 4, we could successfully record evoked inward currents in the odontoblast located 5 μm away from the stimulated odontoblast. The evoked inward currents via intercellular transmitters were likely mediated by the activation of ionotropic receptors and/or ionic channels. Mechanical stimulation of the odontoblasts induces a release of intercellular transmitters, not only of ATP (as in the present study and in Shibukawa et al., 2015), but also of glutamate (our personal communication). However, the present results indicate that the ionotropic receptors (such as ionotropic ATP (P2Xs) and/or glutamate receptors) and ionic channel activation are hardly involved in the inter-odontoblast communication, since the evoked currents elicited by mechanical stimulation could be observed in only 4.8% of the odontoblasts recorded (2/42 cells) in the present study. We previously reported that the P2X_3_ receptor is not expressed in odontoblasts, while the P2X_3_ receptor in the TG neurons plays an important role in the sensory transduction sequence by receiving ATP from mechanically stimulated odontoblasts (Shibukawa et al., 2015). Although, we observed several P2X subtype expressions, except for the P2X_3_ receptor, in odontoblasts (personal communication), these P2X receptor subtypes need a relative high concentration of extracellular ATP to be activated. Therefore, ATP released in response to TRPV1 channel activation during mechanical stimulation predominantly activates P2Y receptors on neighboring odontoblasts to mediate intercellular odontoblasts communication.

We previously showed that accumulated [Ca^2+^]_i_ following TRPV1 channel activation is extruded by Na^+^-Ca^2+^ exchangers (NCXs) in odontoblasts (Tsumura et al., 2012). Our results suggest that communication between odontoblasts mediated by ATP plays an important role in reactionary dentin formation following dentin stimulation by increased intracellular Ca^2+^ signaling by TRPV-mediated Ca^2+^ influx and P2Y receptor-mediated Ca^2+^ mobilization as well as subsequent Ca^2+^ extrusion by NCXs.

## Conclusion

The results from this study show that ATP is released from a mechanically stimulated odontoblast via pannexin-1 by TRP channel activation, and it transmits a signal to nearby odontoblasts by activating P2Y receptors. The results also strongly suggest that odontoblasts communicate with each other to drive their cellular functions such as enhancement of dentin formation. Our results also indicate that odontoblasts are sensory receptor cells (Shibukawa et al., 2015) that detect hydrodynamic force within dentinal tubules (Liu et al., 2015) via mechanosensitive TRP channels. Additionally, ATP released from odontoblasts via pannexin-1 pathway acts as the intercellular transmitter between odontoblasts by activating the P2Y receptors. Taken together with our previous results (Tsumura et al., 2010, 2012, 2013; Sato et al., 2013; Shibukawa et al., 2015), the present study also strongly suggests that the intercellular signaling via released ATP in response to activation of odontoblasts as mechano-sensitive sensory receptor cells are capable of activating two specific and separate pathways underlying odontoblast function: (1) the selective activation of P2Y receptors in odontoblasts establishes inter-odontoblast network that may drive dentin formation, and (2) specific activation of P2X_3_ receptors in TG neurons mediates the sensory transduction sequence for dentin (Shibukawa et al., 2015). In addition, the results also prove that odontoblast cell lines are useful in studying the cellular functions that are mediated by intercellular signaling.

## Author contributions

MS, TF, MK, and YK carried out the measurement intracellular Ca^2+^ signaling. YS, TS, and MT participated in the design of the study. MS and TF performed the statistical analysis. YS conceived of the study, and participated in its design and coordination and helped to draft the manuscript. All authors read and approved the final manuscript.

## Funding

This research was supported by a Grant-in-Aid (No. 26861559/15K11129/15K11056) for Scientific Research from the MEXT of Japan.

### Conflict of interest statement

The authors declare that the research was conducted in the absence of any commercial or financial relationships that could be construed as a potential conflict of interest.
